# Closed-Loop Supply Chain Models with Considering the Environmental Impact

**DOI:** 10.1155/2014/852529

**Published:** 2014-09-16

**Authors:** Amir Mohajeri, Mohammad Fallah

**Affiliations:** ^1^Department of Industrial Engineering, Islamic Azad University, Science and Research Branch, Tehran 14778 93855, Iran; ^2^Department of Industrial Engineering, Islamic Azad University, South Tehran Branch, Tehran 11518 63411, Iran

## Abstract

Global warming and climate changes created by large scale emissions of greenhouse gases are a worldwide concern. Due to this, the issue of green supply chain management has received more attention in the last decade. In this study, a closed-loop logistic concept which serves the purposes of recycling, reuse, and recovery required in a green supply chain is applied to integrate the environmental issues into a traditional logistic system. Here, we formulate a comprehensive closed-loop model for the logistics planning considering profitability and ecological goals. In this way, we can achieve the ecological goal reducing the overall amount of CO_2_ emitted from journeys. Moreover, the profitability criterion can be supported in the cyclic network with the minimum costs and maximum service level. We apply three scenarios and develop problem formulations for each scenario corresponding to the specified regulations and investigate the effect of the regulation on the preferred transport mode and the emissions. To validate the models, some numerical experiments are worked out and a comparative analysis is investigated.

## 1. Introduction

The issue of supply chain management has received increasing attention among the researchers over the last few decades or so. Nowadays, due to the existence of global and competitive market, it is necessary that enterprises work together to enhance their adaptive ability and viability in the market. Hereby, these enterprises achieve common goals such as minimizing the total costs and the delay of deliveries in the whole chain [1–3]. Three main flows exist in the chain: the material flow, the information flow, and the fund flow. Coordination and integration of these flows across enterprises are called a supply chain management (SCM) [4]. The global economic growth from the 20th to the 21st century has led to a rise in consumption of goods. Consequently, large streams of goods all over the world have been founded. In this way, the production and all aspects of logistics such as transportation, warehousing, and inventories have created large environmental problems such as global warming and climate changes [5]. In 1955, an assessment was published by the Intergovernmental Panel on Climate Change (IPCC). This assessment claimed that Earth's surface warming is a result of increase in greenhouse gas concentrations [6, 7]. Greenhouse gases are a collection of gases among which are CO_2_ (carbon dioxide), CH_4_ (methane), N_2_O (nitrous oxide), HFCs (hydrofluorocarbons), PFCs (perfluorocarbons), and SF_6_ (sulphur hexafluoride) [8]. Department of the Environment, Transport and the Regions (DETR) estimated that, among these greenhouse gases, CO_2_ is present in the atmosphere in significant quantities and accounts for two-thirds of global warming [9]. CO_2_ is released from several sources such as transportation, industrial processes, and other commercial sectors. As a result, a greenhouse effect is increased [10]. Integration of SCM concept with the issue of environment protection confirms sharp decline in pollution problem. Research on this approach has received considerable attention recently and led to the creation of new research agenda: green supply chain management (GSCM). So, GSCM is a new paradigm where the supply chain will have a direct relation to the environment. Due to the quality and supply chain revolution in the late 1980s and 1990s, respectively, most enterprises have been motivated to become environmentally conscious and have been faced with pressure to protect the environment in their supply chains [11, 12]. Nowadays, most research on GSCM has had a tendency to the reverse logistics and closed-loop supply chains such as researches done by Blumberg [13] and Pochampally et al. [14]. In the reverse logistics/closed-loop supply chain systems, a product returns to the manufacturer after use and can be repaired or remanufactured to be delivered again to the end consumers. A top environmental issue for an enterprise is how to reduce the utilization of the materials by reusing and remanufacturing the used products. This brings about the GSCM concept and has led to a problem of the closed-loop supply chain management. The closed-loop logistics are divided into two parts. These two parts are given as follows.

- Forward logistics: after manufactory, the distributors are responsible to deliver the final products to the end consumers satisfying their demands.
- Reverse logistics: the flow of used products is processed from the customers back to the dismantlers to do the sorting or disassembling for recovery, reuse, or disposal [13, 14].

With well-managed reverse logistics, the environment protection can be achieved with minimizing of total costs in the whole closed-loop supply chain. Most of the previous studies focused on reverse logistics and only formulated models corresponding to this field. Some researchers presented the closed-loop models, but they did not consider the relation between forward and reverse flows in their proposed models [15–17]. These models often assumed the unlimited capacities for the reverse logistics, which is not a valid assumption for representing the real situations. In real life situations, the DC also plays such role as a collector in a recovery system. So, the capacity of DC is restricted to both distribution and collection. Now, there is an interaction between amounts of the distribution and the collection so that, when the amounts of the collection are larger, the amounts of distribution must decrease under the same capacity. The closed-loop supply chain is characterized by these interactions. With the lack of such kind of relations, the model can be separated into two parts independently and become a supply chain including forward and reverse chains but not a loop. There exist a few studies in which closed-loop models were proposed with realistic assumption. In these studies, researchers shared the same capacity for the reverse logistics and stated the relation between forward and reverse flows [18]. These authors proposed a generalized closed-loop model for the logistics planning. They formulated an integer linear programming model in which the integration between forward and reverse logistics and the decisions for selecting the places such as DCs was considered. Due to NP-hard nature of their model, a genetic algorithm based on spanning tree structure was developed.

Reviewing the literature on closed-loop supply chain, it is concluded that a few studies consider the relations between forward and reverse logistics. In this study, we extend Wang and Hsu's model [18] doing more to protect the environment. First of all, in addition to managing properly reverse logistics to reduce negative impact of greenhouse gases emissions, we suggest another strategy for achieving an expected goal, simultaneously. Here, we focus on a different and important aspect of green supply chains: we focus on transport mode selection as a way to reduce emissions. For this, in addition to minimizing the total cost in the whole closed-loop chain, we consider two types of regulations to reduce carbon emissions coming from freight transport. The first mechanism specifies a cost for carbon emissions and the second one is a constraint on emissions. In this study, we pursue three scenarios and develop problem formulations for each scenario corresponding to these regulations and investigate the effect of the regulation on the preferred transport mode and the emissions. These scenarios are given as follows:

- model without emissions (basic),
- model with emissions (emission-constraint problem),
- model with emissions (emission cost-minimization problem).

Here, we use empirical data to estimate the carbon emissions for various transport modes accurately. The transport modes differ with respect to unit transportation cost, lead time, and unit emissions. For the first scenario in which carbon emissions resulting from freight transport are not considered, our model trades off between long lead times and lower transport costs and short lead times and higher transport costs for transport modes. The carbon emissions are taken into account for the rest of the scenarios where a tradeoff exists between lead time, unit transportation cost, and unit emissions for transport mode. For example, air transport has a shorter lead time, higher unit transportation costs, and carbon emissions than water transport. Defining the three scenarios, we analyze the effect of the regulation on the preferred transport mode and the emissions. Second, we focus on structure of closed-loop supply chain. Many procedures are available in this field. One of these procedures is related to traveling salesman problem (TSP) concept in which, having N cities, a salesman should start from home city, visit all customers once, and come back to the home city finding a minimal route. Multiple traveling salesman problem (mTSP) is a well-known problem in which several salesmen should start and return to a single home city somehow all customers are visited exactly once. Now, we suppose that there are multi-DCs in the proposed supply network. Any of them has a number of salesmen. Multiple DCs, multiple traveling salesmen problem (MDMTSP) finds tours for all salesmen such that all customers are visited exactly once and the total cost of the tours is minimized, while salesmen departure from DCs and arrival to the single destination is called the multiple departures single destination multiple TSP [19]. Third, we consider time windows in our proposed closed-loop supply chain. There are four layers of supply network (manufacturers, DCs, customers, and dismantlers). Customers send order lists and wait to deliver them. The purposes are determination of proper locations of manufactories, DCs, and dismantlers among candidates set and a suitable distribution of goods throughout the network minimizing cost of all tours. Selection of proper manufactories, DCs, and dismantlers to supply customers depends on satisfying time windows on customer's viewpoints. So, embedding the transport mode selection, MDMTSP, and time window concepts in a closed-loop system with respect to the overall amount of CO_2_ emitted from journeys, it is noted that our closed-loop network design is more precisely planned with the aim of protecting environment. To our knowledge, this study is the first paper which considers these concepts simultaneously in the closed-loop supply chain. The remainder of our work is organized as follows. The proposed problem is fully explained and justified in Section 2. The methodology based on empirical data to estimate the carbon emissions for different modes of transport is also discussed. Next, the mathematical formulation for three scenarios is developed. In Section 4, the numerical experiments to illustrate the effectiveness of the proposed methodology are given. A comparative analysis is presented in Section 5. Finally, conclusions are drawn.

## 2. Problem Description

There are essentially four stages along a green logistic network: manufacturers, DCs, customers, and dismantlers. Here, we consider multiple manufactories, DCs, dismantlers, and customers being serviced with one supplier, various transport modes, and one commodity with deterministic demands. The initial problem is making decisions for choosing the proper places of manufactories, DCs, and dismantlers among candidates set while pursuing minimal operations cost, carbon emission, and maximal profits, considering inventory constraints, and satisfying customer demands. Distribution of products from DCs to customers plays critical role. MDMTSP approach can be appropriate for this problem. Any salesman located at DC must depart and visit customers and then go back to the similar or dissimilar DC. In this problem, we suppose that any customer is supplied by only one DC. Meanwhile, the total demands are satisfied. We use the basic conditions for our closed-loop chain and consider them as our assumptions in modeling. These basic conditions are given as follows.

- The customers' demands must be satisfied.
- The flow transferred between two inconsecutive stages must be prevented.
- The number of opened facilities and their capacities are limited.

Recycling rate issue is only discussed in the closed-loop logistics literature. This contains the recovery and landfilling rates. In our model, the recovery amount is assumed to be a percentage of the customer demand corresponding to the Van Der Laan et al. [20] assumption based on the dependence of the amounts of returned products on the demand of the products. So, the following assumption is considered by our model.

(iv) The recovery and landfilling rates are given.

**Figure 1:**
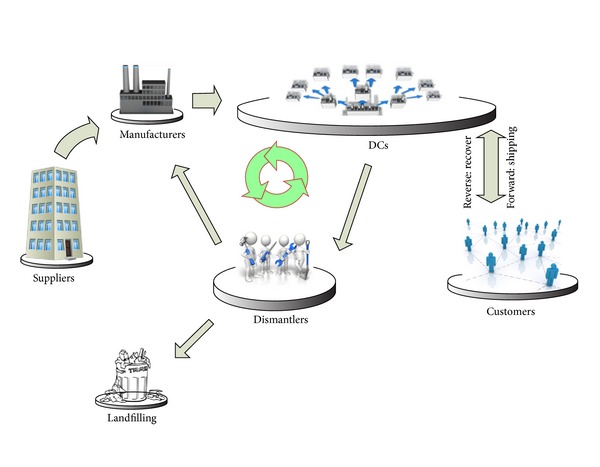
Framework of proposed closed-loop chain.

The framework of proposed closed-loop chain is illustrated in Figure 1. One of the main advantages of our proposed model is integrating the transport mode selection and closed-loop logistics in the supply network. In this study, we design a closed-loop supply chain with the aim of both minimizing the total cost and reducing the environmental impact in the whole chain by choosing the optimal locations of the facilities, the flows of operation units, and the transportation modes along each capacity-constrained stage when the demand of customers and the recycling rates are given. In relation to the transportation issue, it has a significant impact on air pollution so that the overall amount of CO_2_ emitted from it is about 14% of total emissions at the global level [21, 22]. Transportation mode is one of the main choices in transport. There is a variety of transportation modes in our closed-loop chain such as transport by plane, ship, truck, or rail. Costs, transit time, and environmental performance are factors by which each mode is distinguished from other modes. Here, the transport mode is chosen using financial and environmental considerations. Besides, the time window constraints play a key role in selecting the transport mode. Due to the air pollution impacts resulting from freight transportation, this paper pays a special attention to this issue from CO_2_ emission's viewpoint. With respect to the emission calculation issue, there are several methodologies to measure carbon emissions accurately: Greenhouse Gas (GHG) Protocol [23], Artemis [24], EcoTransIT [25], NTM [26], and STREAM [27]. Here, we use the NTM method which specifies emissions for four types of transport: air, rail, road, and water. The NTM method has a high level of detail and focuses on Europe. In this section, we describe the calculation method for the total emissions for each type of transport. This method calculates the total emissions for an average-loaded vehicle and allocates part of the emissions to one unit of product. Below, emissions calculated for four types of transport based on NTM method are given.

*Air Transport.* The emission factor and the distance are the two main elements determining the total emissions coming from the air transport. The emission factor is in two parts: a constant emission factor (CEF) and a variable emission factor (VEF). Estimation of the emission factors from aircraft is based on aircraft type, engine type, and maximum load. With respect to this type of transport, the flight distance (*D*_*a*_) is considered to calculate the distance between the origin and destination location. The bend of the earth is taken into account when we need to calculate the flight distance. The total emissions for an aircraft are calculated by the following equation:

(1) $$\begin{array}{c} {\text{EM}}_{\text{total}}=\text{CEF}+\text{VEF}\cdot D_{a}. \end{array}$$

Defining (1), the total emissions for an average-loaded vehicle have been calculated. If we want to allocate part of the emissions to one unit of product (*e*_*a*_), we also have to define the dimensional weight (*w*_*d*_) which is determined by the density (*ρ*) multiplied by volume (*v*) of one unit of product. Corresponding to [28], if a product has a higher density than 167 (kg/m^3^), the actual weight is considered to calculate the dimensional weight. In contrast to this, the volume multiplied by 167 (kg/m^3^) is substituted for the actual weight when a product has a low density. Then,

(2) $$\begin{array}{c} w_{d}=\max\left(w,167v\right)=\max\left(\rho v,167v\right)=v\max\left(\rho ,167\right). \end{array}$$

Since the amount of goods carried by a vehicle depends on the weight and the volume of the load, the emissions allocated to one unit of the product (*e*_*a*_ in kg) are calculated as follows:

(3) $$\begin{array}{c} e_{a}={\text{EM}}_{\text{total}}\frac{w_{d}}{{\text{LO}}_{\max}\;\text{LF}}, \end{array}$$

where LO_max⁡_ and LF are the maximum load of an aircraft (in kg) and the average load factor of the aircraft, respectively.

*Railway Transport.* Here, the emission calculation method for only diesel engine in railway transportation is described [29]. The unit emissions are calculated (*e*_*d*_) based on the emission factor, the distance, and the weight of the product. The amount of CO_2_ emitted when transporting 1 net tonne over 1 km in way is known as the emission factor (EF in kg CO_2_/net tonne km). It depends on several factors outlined as follows.

- The gross weight of the train (*W*_gr_ in tonne) includes the weight of the locomotive and the carriages.
- An emission constant (*T*) determines the fuel consumption for a way.
- A correcting factor for the terrain (*ξ*_*t*_) is different based on the topography of the way. For example, the factor for* hilly* and* mountainous* terrain is greater than for* flat*. Hence, *ξ*_*f*_ = 1 and *ξ*_*m*_ > *ξ*_*h*_ > 1, where *t* ∈ {flat, mountainous, hilly}.
- The load factor (LF) equals the ratio of net and gross weight of the train.
- The fuel emissions (FE) denote the emissions per liter of fuel burnt.

The emission factor for the diesel rail transport (EF_*d*_ in (kg CO_2_/net tonne km)) is defined by the following equation:

(4) $$\begin{array}{c} \text{E}{\text{F}}_{d}=\frac{\xi_{t}\cdot T\cdot \text{FE}}{10^{6}\sqrt{W_{\text{gr}}}\cdot \text{LF}}. \end{array}$$

The emissions allocated to one unit of the product (*e*_*d*_ in kg) are a function of the distance (*D* in km), the weight of the product (*w* in tonne), and the emission factor. The formula for the unit emissions for the diesel engine in railway transportation is then

(5) $$\begin{array}{c} e_{d}=\text{E}{\text{F}}_{d}\cdot D\cdot w. \end{array}$$

*Road Transport.* In this section, the fuel consumption, the fuel emissions, and the distance are three main factors to calculate the total emissions of the vehicle. Below, each factor is given in more detail.

(i) The fuel consumption (FC in L/km) is based on two factors, load factor (LF) and the type of vehicle, and is calculated as follows:
  (6) $$\begin{array}{c} \text{FC}=\text{F}{\text{C}}_{\text{empty}}+\left(\text{F}{\text{C}}_{\text{full}}-\text{F}{\text{C}}_{\text{empty}}\right)\cdot \text{LF}, \end{array}$$

where FC_full_ and FC_empty_ are the fuel consumption for a full loaded vehicle and the fuel consumption for an unladen vehicle, respectively.

(ii) The fuel emissions (FE) are defined as gram of CO_2_ emitted per liter of fuel.
(iii) The distance (*D* in km) is the distance between the locations.

Combining these factors yields the following equation for the total emissions of the vehicle for road transport (EM_total_ in g):

(7) $$\begin{array}{c} {\text{EM}}_{\text{total}}=\text{FE}\cdot \text{FC}\cdot D. \end{array}$$

Defining (7), the emissions of the entire vehicle have been calculated. If we want to allocate part of the emissions to one unit of product (*e*_*r*_), we also have to define the dimensional weight (*w*_*d*_) of one unit of product, which is defined as

(8) $$\begin{array}{c} w_{d}=\max\left(w,250v\right)=\max\left(\rho v,250v\right)=v\max\left(\rho ,250\right), \end{array}$$

where 250 is a default density used by transport companies [30]. So, if a product has a density higher than 250 (kg/m^3^), the actual weight is considered to calculate the dimensional weight. In contrast to this, the volume multiplied by 250 (kg/m^3^) is substituted for the actual weight when a product has a low density. The emissions allocated to one unit of the product (*e*_*r*_ in g) are calculated as follows:

(9) $$\begin{array}{c} e_{r}={\text{EM}}_{\text{total}}\frac{w_{d}}{{\text{LO}}_{\max}\text{LF}}, \end{array}$$

where LO_max⁡_ and LF are the maximum load of a vehicle (in kg) and the average load factor of the vehicle, respectively.

*Water Transport.* Short-sea transport with diesel oil-powered vessels is known as water transport [31]. Here, the total emissions (EM_total_ in kg) depend on three factors: the fuel consumption (FC), the fuel emissions (FE), and the distance (*D*_*w*_). The fuel consumption (FC) (in L per km) is given in [31] for both a given vessel type and an average load factor. The distance *D*_*w*_ (in km) is the distance between two locations over waterways which is larger than the distance over road. The fuel emissions (FE) factor (in kg) is also the amount of CO_2_ emitted when 1 L of diesel is burnt. The total emissions (EM_total_ in kg) of the vessel are calculated by the following equation:

(10) $$\begin{array}{c} {\text{EM}}_{\text{total}}=\text{FE}\cdot \text{FC}\cdot D_{w}. \end{array}$$

The unit emissions for the vessel in waterway transportation are obtained defining the allocation fraction *α* ∈ (0, 1] as follows:

(11) $$\begin{array}{c} \alpha =\frac{\text{unit}\;\text{capacity}}{\text{total}\;\text{capacity}}, \end{array}$$

where the type of ship plays a critical role in determining the unit of capacity; here, it can be weight for bulk vessels. The formula for the unit emissions (*e*_*w*_ in kg) of the vessel is then

(12) $$\begin{array}{c} e_{w}=\alpha \cdot {\text{EM}}_{\text{total}}=\alpha \cdot \text{FE}\cdot \text{FC}\cdot D_{w}. \end{array}$$

## 3. Supply Chain Models with Considering the Environmental Impact

Here, we pursue three scenarios and develop problem formulations for each scenario. A mixed integer linear programming (MILP) optimization model to minimize the total construction cost of this network is presented.

### 3.1. Basic Model

In order to simplify this problem, we suppose that there is only one product in the concerned closed-loop chain, and the carbon emission resulting from freight transport is not considered. In order to formulate this simplified problem mathematically, the following notations are necessary.

*Notations*

 
*I*: Set of candidate manufactories
 
*J*: Set of candidate DCs
 
*K*: Set of customers
 
*M*: Set of candidate dismantlers
 
*V*: Set of transport mode types
 
*V*_*I*_: Set of transport mode types at manufactory; *V*_*I*_ ⊂ *V*
 
*V*_*J*_: Set of transport mode types at DC; *V*_*J*_ ⊂ *V*
 
*V*_*M*_: Set of transport mode types at dismantler; *V*_*M*_ ⊂ *V*.

*Parameters*

 Cm_*i*_: Capacity of manufactory *i*
 Tc_*j*_: Total capacity of DC*j* (forward and reverse)
 Cd_*m*_: Capacity of dismantler *m*
 Pc_*j*_: The percentage of total capacity for reverse logistics in DC*j*
 pr_*k*_: The percentage of recovery of customer *k*
 Pl_*m*_: The percentage of landfilling of dismantler *m*
 dc_*k*_: Demand of customer *k*
 
*P*_cost_*i*_: Unit cost of production in manufactory *i*
 CMD_*v*_*i*__: Unit cost of transportation from manufactory to DC by vehicle *v*_*i*_ per km
 CDC_*v*_*j*__: Unit cost of transportation from DC to customer by vehicle *v*_*j*_ per km
 CDM_*v*_*m*__: Unit cost of transportation from dismantler to manufactory by vehicle *v*_*m*_ per km
 FM_*i*_: Fixed cost for operating manufactory *i*
 FDC_*j*_: Fixed cost for operating DC*j*
 FD_*m*_: Fixed cost for operating dismantler *m*
 Cl: Fixed cost for landfilling per unit
 dis_MD_*ij*_: Distance between manufactory *i* and DC*j*
 dis_DC_*jk*_: Distance between DC*j* and customer *k*
 dis_CC_*kl*_: Distance between customer *k* and customer *l*
 dis_DD_*jm*_: Distance between DC*j* and dismantler *m*
 dis_DM_*mi*_: Distance between dismantler *m* and manufactory *i*
 
*t*_DC_*jkv*_*j*__: The time of transportation from DC*j* to customer *k* using vehicle *v*_*j*_
 
*t*_CC_*klv*_*j*__: The time of transportation from customer *k* to customer *l* using vehicle *v*_*j*_
 
*a*_*c*_*k*_: The lower bound of expected time for delivering product at customer *k*
 
*b*_*c*_*k*_: The upper bound of expected time for delivering product at customer *k*
 Rc_*kj*_: The recovery cost in DC*j* from customer *k*
 NVM_*iv*_*i*__: Number of vehicles *v*_*i*_ at manufactory *i*
 NVD_*jv*_*j*__: Number of vehicles *v*_*j*_ at DC*j*
 NVDi_*mv*_*m*__: Number of vehicles *v*_*m*_ at dismantler *m*
 CVM_*v*_*i*__: Capacity of vehicle *v*_*i*_
 CVD_*v*_*j*__: Capacity of vehicle *v*_*j*_
 CVDi_*v*_*m*__: Capacity of vehicle *v*_*m*_
 LO_max⁡__*M*_*v*_*i*__: Maximum load for vehicle *v*_*i*_
 LO_max⁡__*D*_*v*_*j*__: Maximum load for vehicle *v*_*j*_
 LO_max⁡__Di_*v*_*m*__: Maximum load for vehicle *v*_*m*_
 LF_*M*_*v*_*i*__: Average load factor for vehicle *v*_*i*_
 LF_*D*_*v*_*j*__: Average load factor for vehicle *v*_*j*_
 LF_Di_*v*_*m*__: Average load factor for vehicle *v*_*m*_
 vol: Volume of product
 
*ρ*_*v*_*i*__: Density of product for vehicle *v*_*i*_
 wp: Weight of product
 capw: Total capacity of cargo vessel
 
*Q*: The maximum number of nodes a salesman may visit
 
*L*: The minimum number of nodes a salesman must visit
 
*M*: A large number.

*Decision Variables*

 
*x*_MD_*ijv*_*i*__: 1, if a product can be shipped by vehicle *v*_*i*_ from manufactory *i* to DC*j*; 0, otherwise
 
*x*_DC_*jkv*_*j*__: 1, if a product can be shipped by vehicle *v*_*j*_ from DC*j* to customer *k*; 0, otherwise
 
*x*_DD_*jmv*_*j*__: 1, if a recovered product can be shipped by vehicle *v*_*j*_ from DC*j* to dismantler *m*; 0, otherwise
 
*x*_CD_*kjv*_*j*__: 1, if a vehicle *v*_*j*_ returned from customer *k* to DC*j*; 0, otherwise
 
*x*_DM_*miv*_*m*__: 1, if a reused product can be shipped by vehicle *v*_*m*_ from dismantler *m* to manufactory *i*; 0, otherwise
 
*α*_*i*_: 1, if production takes place on manufactory *i*; 0, otherwise
 
*β*_*j*_: 1, if DC*j* is opened; 0, otherwise
 
*γ*_*m*_: 1, if dismantler *m* is opened; 0, otherwise
 
*z*_CC_*klv*_*j*__: 1, if a product can be shipped by vehicle *v*_*j*_ from customer *k* to customer *l*; 0, otherwise
 
*y*_MD_*ijv*_*i*__: Amount shipped by vehicle *v*_*i*_ from manufactory *i* to DC*j*
 
*y*_DC_*jkv*_*j*__: Amount shipped by vehicle *v*_*j*_ from DC*j* to customer *k*
 
*y*_DD_*jmv*_*j*__: Amount of recovered product shipped by vehicle *v*_*j*_ from DC*j* to dismantler *m*
 
*y*_CD_*kjv*_*j*__: Recovered amount shipped by vehicle *v*_*j*_ from customer *k* to DC*j*
 
*y*_DM_*miv*_*m*__: Reused amount shipped by vehicle *v*_*m*_ from dismantler *m* to manufactory *i*
 
*y*_CC_*klv*_*j*__: Recovered amount shipped by vehicle *v*_*j*_ from customer *k* to customer *l*
 
*PM*_*i*_: Quantity produced at manufactory *i*
 
*u*_*k*_: The number of nodes visited by travelers from DC to node *k*
 cong*R*_*k*_: Amount of congested product at customer *k*
 cong*F*_*k*_: Amount of congested recovered product at customer *k*
 
*S*_*k*_: The arrival time of product at customer *k*.

Using these definitions, the basic model for the proposed closed-loop chain can be described as follows.

*Objective Function*

Consider the following:

(13) f=∑i∈Iαi·FMi+∑j∈Jβj·FDCj+∑m∈Mγm·FDm+∑i∈I ∑j∈J ∑vi∈VIy_MDijvi·dis_MDij·CMDvi+∑j∈J ∑k∈K ∑vj∈VJy_DCjkvj·dis_DCjk·CDCvj+∑k∈K ∑l∈K ∑vj∈VJy_CCklvj·dis_CCkl·CDCvj+∑k∈K ∑j∈J ∑vj∈VJy_CDkjvj·dis_DCjk·CDCvj+∑j∈J ∑m∈M ∑vj∈VJy_DDjmvj·dis_DDjm·CDCvj+∑m∈M ∑i∈I ∑vm∈VMy_DMmivm·dis_DMmi·CDMvm+∑i∈IPMi·P_costi+∑k∈K ∑j∈J ∑vj∈VJy_CDkjvj·Rckj+CL·∑m∈M⌊Plm·∑j∈J ∑vj∈VJy_DDjmvj⌋.

*Constraints*

Consider the following:

(14) $$\begin{array}{c} \sum_{i\in I}\alpha_{i}\geq 1, \end{array}$$

(15) $$\begin{array}{c} \sum_{j\in J}\beta_{j}\geq 1, \end{array}$$

(16) $$\begin{array}{c} \text{P}{\text{M}}_{i}\geq 1-M\left(1-\alpha_{i}\right),\;\forall i\in I, \end{array}$$

(17) $$\begin{array}{c} \sum_{v_{i}\in V_{I}}\;\sum_{j\in J}x\_\text{M}{\text{D}}_{ijv_{i}}\geq 1-M\left(1-\alpha_{i}\right),\;\forall i\in I, \end{array}$$

(18) $$\begin{array}{c} \sum_{v_{i}\in V_{I}}\sum_{i\in I}x\_\text{M}{\text{D}}_{ijv_{i}}\geq 1-M\left(1-\beta_{j}\right),\;\forall j\in J, \end{array}$$

(19) y_MDijvi≥1−M(1−x_MDijvi),∀i∈I, ∀j∈J, ∀vi∈VI,

(20) $$\begin{array}{c} \sum_{v_{i}\in V_{I}}\;\sum_{j\in J}y\_\text{M}{\text{D}}_{ijv_{i}}\leq \text{C}{\text{m}}_{i},\;\forall i\in I, \end{array}$$

(21) $$\begin{array}{c} \sum_{j\in J}x\_\text{M}{\text{D}}_{ijv_{i}}\leq \text{NV}{\text{M}}_{iv_{i}},\;\forall i\in I,\;\;\forall v_{i}\in V_{I}, \end{array}$$

(22) $$\begin{array}{c} \sum_{v_{i}\in V_{I}}x\_\text{M}{\text{D}}_{ijv_{i}}\leq 1,\;\forall i\in I,\;\;\forall j\in J, \end{array}$$

(23) wp·y_MDijvi≤CVMvi·LF_Mvi,∀i∈I, ∀j∈J, ∀vi∈VI,

(24) $$\begin{array}{c} \sum_{v_{j}\in V_{J}}\sum_{k\in K}x\_\text{D}{\text{C}}_{jkv_{j}}\geq 1-M\left(1-\beta_{j}\right),\;\forall j\in J, \end{array}$$

(25) $$\begin{array}{c} \sum_{v_{j}\in V_{J}}\sum_{k\in K}x\_\text{C}{\text{D}}_{kjv_{j}}\geq 1-M\left(1-\beta_{j}\right),\;\forall j\in J, \end{array}$$

(26) $$\begin{array}{c} \sum_{v_{j}\in V_{J}}\;\sum_{j\in J}x\_\text{D}{\text{C}}_{jkv_{j}}+\sum_{v_{j}\in V_{J}}\;\sum_{j\in J}x\_\text{C}{\text{D}}_{kjv_{j}}\leq 1,\;\forall k\in K, \end{array}$$

(27) $$\begin{array}{c} \sum_{v_{j}\in V_{J}}\;\sum_{j\in J}x\_\text{D}{\text{C}}_{jkv_{j}}+\sum_{v_{j}\in V_{J}}\;\sum_{l\in k}z\_\text{C}{\text{C}}_{lkv_{j}}=1,\;\forall k\in K, \end{array}$$

(28) $$\begin{array}{c} \sum_{v_{j}\in V_{J}}\;\sum_{j\in J}x\_\text{C}{\text{D}}_{kjv_{j}}+\sum_{v_{j}\in V_{J}}\;\sum_{l\in k}z\_\text{C}{\text{C}}_{klv_{j}}=1,\;\forall k\in K, \end{array}$$

(29) ∑l∈Kz_CCklvj+∑j∈Jx_CDkjvj=∑e∈Kz_CCekvj+∑j∈Jx_DCjkvj,∀k∈K, ∀vj∈VJ,

(30) u(k)−u(l)+(Q·z_CCklvj)+((Q−2)·z_CClkvj) ≤Q−1, ∀k,l∈K,∀vj∈VJ,

(31) u(k)+((Q−2)·∑vj∈Vj ∑j∈Jx_DCjkvj)−∑vj∈VJ ∑j∈Jx_CDkjvj ≤Q−1, ∀k∈K,

(32) ∑vj∈VJ ∑j∈Jx_DCjkvj+((2−L)·∑vj∈VJ∑j∈Jx_CDkjvj)≥2,∀k∈K,

(33) $$\begin{array}{c} \text{cong}R_{k}=\left(\sum_{v_{j}\in V_{J}}\;\sum_{l\in k}z\_\text{C}{\text{C}}_{klv_{j}}\cdot \text{cong}R_{l}\right)+\text{d}{\text{c}}_{k},\;\forall k\in K, \end{array}$$

(34) y_DCjkvj≥1−M(1−x_DCjkvj),∀k∈K, ∀vj∈VJ, ∀j∈J,

(35) $$\begin{array}{c} y\_\text{D}{\text{C}}_{jkv_{j}}\geq \text{cong}R_{k},\;\forall k\in K,\;\;\forall v_{j}\in V_{J},\;\;\forall j\in J, \end{array}$$

(36) wp·y_DCjkvj≤CVDvj·LF_Dvj,∀k∈K, ∀vj∈VJ, ∀j∈J,

(37) $$\begin{array}{c} \sum_{k\in K}x\_\text{D}{\text{C}}_{jkv_{j}}\leq \text{NV}{\text{D}}_{jv_{j}},\;\forall v_{j}\in V_{J},\;\;\forall j\in J, \end{array}$$

(38) $$\begin{array}{c} \sum_{v_{i}\in V_{I}}\;\sum_{i\in I}y\_\text{M}{\text{D}}_{ijv_{i}}=\sum_{v_{j}\in V_{J}}\;\sum_{k\in K}y\_\text{D}{\text{C}}_{jkv_{j}},\;\forall j\in J, \end{array}$$

(39) congFk=(∑vj∈VJ∑l∈kz_CClkvj·congFl)+⌈prk·dck⌉,∀k∈K,

(40) y_CDkjvj≥1−M(1−x_CDkjvj),∀k∈K, ∀vj∈VJ, ∀j∈J,

(41) $$\begin{array}{c} y\_\text{C}{\text{D}}_{kjv_{j}}\geq \text{cong}F_{k},\;\forall k\in K,\;\;\forall v_{j}\in V_{J},\;\;\forall j\in J, \end{array}$$

(42) y_CCklvj≥(∑j∈Jy_DCjkvj+∑h∈Ky_CChkvj) −⌈(1−prk)·dck⌉−M(1−z_CCklvj),∀k,l∈K, ∀vj∈VJ,

(43) $$\begin{array}{c} \sum_{v_{j}\in V_{J}}\;\sum_{j\in J}x\_\text{D}{\text{D}}_{jmv_{j}}\geq 1-M\left(1-\gamma_{m}\right),\;\forall m\in M, \end{array}$$

(44) $$\begin{array}{c} \sum_{v_{j}\in V_{J}}x\_\text{D}{\text{D}}_{jmv_{j}}\leq 1,\;\forall j\in J,\;\;\forall m\in M, \end{array}$$

(45) $$\begin{array}{c} \sum_{v_{j}\in V_{J}}\sum_{m\in M}x\_\text{D}{\text{D}}_{jmv_{j}}\geq 1-M\left(1-\beta_{j}\right),\;\forall j\in J, \end{array}$$

(46) y_DDjmvj≥1−M(1−x_DDjmvj),∀j∈J, ∀m∈M, ∀vj∈VJ,

(47) $$\begin{array}{c} \sum_{v_{j}\in V_{J}}\;\sum_{k\in K}y\_\text{C}{\text{D}}_{kjv_{j}}=\sum_{v_{j}\in V_{J}}\;\sum_{m\in M}y\_\text{D}{\text{D}}_{jmv_{j}},\;\forall j\in J, \end{array}$$

(48) ∑vj∈VJ ∑k∈Ky_DCjkvj+∑vj∈VJ ∑m∈My_DDjmvj≤Tcj·βj,∀j∈J,

(49) $$\begin{array}{c} \sum_{m\in M}x\_\text{D}{\text{D}}_{jmv_{j}}\leq \text{NV}{\text{D}}_{jv_{j}},\;\forall j\in J,\;\;\forall v_{j}\in V_{J}, \end{array}$$

(50) wp·y_DDjmvj≤CVDvj·LF_Dvj,∀j∈J, ∀m∈M, ∀vj∈VJ,

(51) $$\begin{array}{c} \sum_{v_{j}\in V_{J}}\;\sum_{m\in M}y\_\text{D}{\text{D}}_{jmv_{j}}\leq \left\lfloor \text{P}{\text{c}}_{j}\cdot \text{T}{\text{c}}_{j}\cdot \beta_{j}\right\rfloor ,\;\forall j\in J, \end{array}$$

(52) $$\begin{array}{c} \sum_{v_{m}\in V_{m}}\;\sum_{i\in I}x\_\text{D}{\text{M}}_{miv_{m}}\geq 1-M\left(1-\gamma_{m}\right),\;\forall m\in M, \end{array}$$

(53) $$\begin{array}{c} \sum_{v_{m}\in V_{M}}x\_\text{D}{\text{M}}_{miv_{m}}\leq 1,\;\forall m\in M,\;\;\forall i\in I, \end{array}$$

(54) $$\begin{array}{c} \sum_{v_{m}\in V_{m}}\;\sum_{m\in M}x\_\text{D}{\text{M}}_{miv_{m}}\geq 1-M\left(1-\alpha_{i}\right),\;\forall i\in I, \end{array}$$

(55) y_DMmivm≥1−M(1−x_DMmivm),∀m∈M, ∀i∈I, ∀vm∈VM,

(56) ∑vm∈VM ∑m∈My_DMmivm+PMi=∑vi∈Vi ∑j∈Jy_MDijvi,∀i∈I,

(57) ∑vj∈VJ ∑j∈Jy_DDjmvj=⌊Plm·∑vj∈VJ ∑j∈Jy_DDjmvj⌋ +∑vm∈VM∑i∈Iy_DMmivm, ∀m∈M,

(58) ∑vm∈VM∑i∈Iy_DMmivm+⌊Plm·∑vj∈VJ∑j∈Jy_DDjmvj⌋  ≤γm·Cdm, ∀m∈M,

(59) $$\begin{array}{c} \sum_{i\in I}x\_\text{D}{\text{M}}_{miv_{m}}\leq \text{NVD}{\text{i}}_{mv_{m}},\;\forall m\in M,\;\;\forall v_{m}\in V_{M}, \end{array}$$

(60) wp·y_DMmivm≤CVDivm·LF_Divm,∀m∈M, ∀i∈I, ∀vm∈VM,

(61) $$\begin{array}{c} S_{k}\geq a\_c_{k},\;\forall k\in K, \end{array}$$

(62) $$\begin{array}{c} S_{k}\leq b\_c_{k},\;\forall k\in K, \end{array}$$

(63) Sk+t_CCklvj−M(1−z_CCklvj)≤Sl,∀k,l∈K, ∀vj∈VJ,

(64) Sk+t_CCklvj+M(1−z_CCklvj)≥Sl,∀k,l∈K, ∀vj∈VJ,

(65) t_DCjkvj−M(1−x_DCjkvj)≤Sk,∀k∈K, ∀vj∈VJ, ∀j∈J,

(66) t_DCjkvj+M(1−x_DCjkvj)≥Sk,∀k∈K, ∀vj∈VJ, ∀j∈J,

(67) x_MDijvi,  x_DCjkvj,  x_DDjmvj,  x_CDkjvj,x_DMmivm,  z_CCklvj,  αi,βj,γm∈{0,1},∀i∈I, ∀j∈J, ∀k,l∈K, ∀m∈M,∀vi∈VI, ∀vj∈VJ, ∀vm∈VM,

(68) y_MDijvi,y_DCjkvj,y_DDjmvj,y_CDkjvj,y_DMmivm,y_CCklvj,PMi,uk,congRk,congFk,Sk≥0,∀i∈I, ∀j∈J, ∀k,l∈K, ∀m∈M,∀vi∈VI, ∀vj∈VJ, ∀vm∈VM.

Equation (13) is the objective function which minimizes cost of opening manufactory, distribution center, and dismantler, minimizes the total cost of both forward and backward distances, and minimizes the total cost of operations. Constraints (14) and (15) show that there exist at least one activated manufactory and one DC in the chain, respectively. Constraint (16) ensures that each manufactory can produce an amount of product just after it is selected. Each activated manufactory covers at least one DC, and Constraint (17) represents this goal. On the contrary, each DC receives at least one link from manufactories just after it is selected (Constraint (18)). Constraint (19) represents the amount of flow between manufactory and DC. Constraint (20) represents the limit of the capacity for manufactories in forward logistics. Constraint (21) imposes that the number of traveling vehicles from manufactory would not exceed the existing vehicles. Constraint (22) prevents the route between manufactory and DC from accepting its vehicle more than once. The capacity constraint of each vehicle that traveled from manufactory to DC is shown by Constraint (23). Constraint (24) guarantees that each activated DC covers at least one customer. Each activated DC receives at least one link from customers, and Constraint (25) represents this goal. Constraint (26) represents that a salesman from DC must visit at least two customers. Constraint (27) requires that any customer be supplied by either DC or other customers. In addition, it either comes back to DC or supplies other customers. This concept is represented by Constraint (28). Each customer is supplied and supplies by the same vehicle. This is represented by Constraint (29). Constraints (30), (31), and (32) prevent any subtour in network. Constraint (33) indicates the amount of congested product for supplying other customers by each customer. Constraint (34) represents the amount of flow between DC and customer. Constraint (35) is to satisfy the customer demand. The capacity constraint of each vehicle traveling from DC to customer is shown by Constraint (36). Constraint (37) imposes that the number of traveling vehicles from DC would not exceed the existing vehicles. Constraint (38) satisfies the law of the flow conservation by in-flow equal to out-flow. The amount of congested product for recovering from other customers by each customer is indicated by Constraint (39). Constraints (40)-(41) represent the amount of flow between customer and DC. The amount of flow among customers is represented by Constraint (42). Constraint (43) guarantees that each activated dismantler receives at least one link from DCs. Constraint (44) prevents the route between DC and dismantler from accepting its vehicle more than once. Constraint (45) guarantees that each activated DC covers at least one dismantler. The amount of flow between DC and dismantler is shown by Constraint (46). Constraint (47) satisfies the law of the flow conservation by in-flow equal to out-flow. Constraint (48) indicates that the total forward and backward flows cannot exceed the total capacity of DC. Constraint (49) imposes that the number of traveling vehicles from DC to dismantler would not exceed the existing vehicles. The capacity constraint of each vehicle traveling from DC to dismantler is shown by Constraint (50). Constraint (51) means the reverse limit of the capacity for DCs. Constraint (52) ensures that each activated dismantler covers at least one manufactory. Constraint (53) prevents the route between dismantler and manufactory from accepting its vehicle more than once. Constraint (54) guarantees that each activated manufactory receives at least one link from dismantlers. The amount of flow between dismantler and manufactory is shown by Constraint (55). Constraints (56) and (57) satisfy the law of the flow conservation by in-flow equal to out-flow. Constraint (58) means the reverse limit of the capacity for dismantlers. Constraint (59) imposes that the number of traveling vehicles from dismantler to manufactory would not exceed the existing vehicles. The capacity constraint of each vehicle traveling from dismantler to manufactory is shown by Constraint (60). Constraints (61)–(66) satisfy time windows. Constraint (67) denotes the binary variables, and Constraint (68) restricts all other variables from taking nonnegative values.

#### 3.1.1. Linearization

To improve the performance of the proposed mathematical model we act out the following linearization for the nonlinear equations. As Constraint (33) is nonlinear, we turn it into the following equations:

(69) Equation  (33)⟶congRk≥M·(z_CCklvj−1)+(dck+congRl),∀vj∈VJ, ∀l,k∈K,

(70)  congRk≤(−M)·(z_CCklvj−1)+(dck+congRl),∀vj∈VJ, ∀l,k∈K,

(71) $$\begin{array}{c} \;\;\text{cong}R_{k}\leq \left(\sum_{v_{j}\in V_{J}}\;\sum_{l\in k}z\_\text{C}{\text{C}}_{klv_{j}}\right)\cdot M+\text{d}{\text{c}}_{k},\;\forall k\in K, \end{array}$$

(72)    congRk≥(∑vj∈VJ ∑l∈kz_CCklvj)·(−M)+dck,∀k∈K.

As Constraint (39) is nonlinear, we turn it into the following equations:

(73) Equation  (39)⟶congFk≥M·(z_CCklvj−1)+((prk·dck)+congFl),∀vj∈VJ, ∀l,k∈K,

(74)  congFk≤(−M)·(z_CCklvj−1)+((prk·dck)+congFl),∀vj∈VJ, ∀l,k∈K,

(75)  congFk≤(∑vj∈VJ ∑l∈kz_CCklvj)·M+(prk·dck),∀k∈K,

(76)  congFk≥(∑vj∈VJ∑l∈kz_CCklvj)·(−M)+(prk·dck),∀k∈K.

### 3.2. Emissions

Here, we describe how the carbon emissions are incorporated into our model and the methodology to calculate the emissions. In Section 3.2.1 we define the emission cost-minimization problem in which a unit cost for emission is charged. In Section 3.2.2 we define the emission-constraint problem in which we have a hard constraint on the carbon emissions.

#### 3.2.1. Emission Cost-Minimization Model

The objective of the proposed basic model is to minimize the total construction and operations costs while considering structural, product flow, capacity, customers' demands, and time windows constraints. It has ignored the carbon emission as an important factor for green supply chain. Below, we will extend the basic model by adding a cost for carbon emissions. In the Emission Trading Scheme the carbon cost is expressed in €/(metric) tonne emissions. We therefore specify a carbon emission cost CE  (CE > 0) per tonne of CO_2_ emitted. For any transportation mode, let EM_total_MD_*ij*_ and *e*_*u*_MD_*ij*_, EM_total_DC_*jK*_ and *e*_*u*_DC_*jk*_, EM_total_CC_*Kl*_ and *e*_*u*_CC_*kl*_, EM_total_CD_*Kj*_ and *e*_*u*_CD_*kj*_, EM_total_DD_*jm*_ and *e*_*u*_DD_*jm*_, and EM_total_DM_*mi*_ and *e*_*u*_DM_*mi*_ denote its total carbon emissions and the emissions allocated to one unit of the product for transportation from the *i*th manufactory to the *j*th DC, from the *j*th DC to the *k*th customer, from the *k*th customer to the *l*th customer, from the *k*th customer to the *j*th DC, from the *j*th DC to the *m*th dismantler, and from the *m*th dismantler to the *i*th manufactory, respectively. In this model, the following objectives and constraints are added to the proposed basic closed-loop model to consider the carbon emission issue. In order to formulate this emission cost-minimization model mathematically, the following notations are necessary.

*Parameters*

 CEF: Constant emission factor
 VEF: Variable emission factor
 FC_*D*_*v*_*j*__: The fuel consumption for vehicle *v*_*j*_
 FE_*D*_*v*_*j*__: The fuel emissions for diesel fuel for vehicle *v*_*j*_
 FC_*M*: The fuel consumption for semitrailer stated in manufactory
 FE_*M*: The fuel emissions for diesel fuel for semitrailer stated in manufactory
 FC_Di: The fuel consumption for semitrailer stated in dismantler
 FE_Di: The fuel emissions for diesel fuel for semitrailer stated in dismantler
 
*T*: The fuel consumption factor for diesel train
 FER: The fuel emissions for diesel train
 
*W*_gr: The gross weight of the train
 FEW: The fuel emissions for cargo vessel
 FCW: The fuel consumption for cargo vessel
 CE: The price of carbon emission (expressed in €/(metric) tonne emissions).

*Decision Variables*

 EM_total_MD_*ij*_: Total emissions of the vehicle from manufactory *i* to DC*j*
 EM_total_DC_*jk*_: Total emissions of the vehicle from DC*j* to customer *k*
 EM_total_CD_*kj*_: Total emissions of the vehicle from customer *k* to DC*j*
 EM_total_CC_*kl*_: Total emissions of the vehicle from customer *k* to customer *l*
 EM_total_DD_*jm*_: Total emissions of the vehicle from DC*j* to dismantler *m*
 EM_total_DM_*mi*_: Total emissions of the vehicle from dismantler *m* to manufactory *i*
 
*e*_*u*_MD_*ij*_: Unit emissions of the vehicle from manufactory *i* to DC*j*
 
*e*_*u*_DC_*jk*_: Unit emissions of the vehicle from DC*j* to customer *k*
 
*e*_*u*_CD_*kj*_: Unit emissions of the vehicle from customer *k* to DC*j*
 
*e*_*u*_CC_*kl*_: Unit emissions of the vehicle from customer *k* to customer *l*
 
*e*_*u*_DD_*jm*_: Unit emissions of the vehicle from DC*j* to dismantler *m*
 
*e*_*u*_DM_*mi*_: Unit emissions of the vehicle from dismantler *m* to manufactory *i*.

*Objective Function*

Consider the following:

(77) f′=∑i∈I ∑j∈J ∑vi∈VIy_MDijvi·e_u_MDij·CE+∑j∈J ∑k∈K ∑vj∈VJy_DCjkvj·e_u_DCjk·CE+∑k∈K ∑l∈K ∑vj∈VJy_CCklvj·e_u_CCkl·CE+∑k∈K ∑j∈J ∑vj∈VJy_CDkjvj·e_u_CDkj·CE+∑j∈J ∑m∈M ∑vj∈VJy_DDjmvj·e_u_DDjm·CE+∑m∈M ∑i∈I ∑vm∈VMy_DMmivm·e_u_DMmi·CE.

*Constraints*

Consider the following:

(78) EM_total_MDij≥(CEF+( VEF·0.801  dis_MDij)) −M(1−x_MDija) −M(x_MDijr+x_MDijt+x_MDijw),∀i∈I, ∀j∈J,

(79) e_u_MDij≥((v·ρa·EM_total_MDij)(LOmax⁡_Ma·LF_Ma))−M(1−x_MDija)−M(x_MDijr+x_MDijt+x_MDijw),∀i∈I, ∀j∈J,

(80) EM_total_MDij≥(FE_M·FC_M·(dis_MDij))−M(1−x_MDijr)−M(x_MDija+x_MDijt+x_MDijw),∀i∈I, ∀j∈J,

(81) e_u_MDij≥((v·ρr·EM_total_MDij)(LOmax⁡_Mr·LF_Mr))−M(1−x_MDijr)−M(x_MDija+x_MDijt+x_MDijw),∀i∈I, ∀j∈J,

(82) EM_total_MDij≥(10−3·(ξf·T·FER)106(W_gr·LF_Mt))−M(1−x_MDijt)−M(x_MDija+x_MDijr+x_MDijw),∀i∈I, ∀j∈J,

(83) e_u_MDij≥(EM_total_MDij·dis_MDij·wp)−M(1−x_MDijt)−M(x_MDija+x_MDijr+x_MDijw),∀i∈I, ∀j∈J,

(84) EM_total_MDij≥(FCW·FEW·1.2dis_MDij)−M(1−x_MDijw)−M(x_MDija+x_MDijr+x_MDijt),∀i∈I, ∀j∈J,

(85) e_u_MDij≥((wp·EM_total_MDij)(capw·1000))−M(1−x_MDijw)−M(x_MDija+x_MDijr+x_MDijt),∀i∈I, ∀j∈J,

(86) EM_total_DCjK≥(FE_Dvj·FC_Dvj·(dis_DCjk))−M(1−x_DCjkvj),∀k∈K, ∀j∈J, ∀vj∈VJ,

(87) e_u_DCjk≥((v·ρr·EM_total_DCjk)(LOmax⁡_Dvj·LF_Dvj))−M(1−x_DCjkvj),∀k∈K, ∀j∈J, ∀vj∈VJ,

(88) EM_total_CCKl≥(FE_Dvj·FC_Dvj·(dis_CCkl))−M(1−z_CCklvj),∀k,l∈K, ∀vj∈VJ,

(89) e_u_CCkl≥((v·ρr·EM_total_CCkl)(LOmax⁡_Dvj·LF_Dvj))−M(1−z_CCklvj),∀k,l∈K, ∀vj∈VJ,

(90) EM_total_CDKj≥(FE_Dvj·FC_Dvj·(dis_DCjk))−M(1−x_CDkjvj),∀k∈K, ∀j∈J, ∀vj∈VJ,

(91) e_u_CDkj≥((v·ρr·EM_total_CDkj)(LOmax⁡_Dvj·LF_Dvj))−M(1−x_CDkjvj),∀k∈K, ∀j∈J, ∀vj∈VJ,

(92) EM_total_DDjm≥(FE_Dvj·FC_Dvj·(dis_DDjm))−M(1−x_DDjmvj),∀m∈M, ∀j∈J, ∀vj∈VJ,

(93) e_u_DDjm≥((v·ρr·EM_total_DDjm)(LOmax⁡_Dvj·LF_Dvj)) −M(1−x_DDjmvj),  ∀m∈M, ∀j∈J, ∀vj∈VJ,

(94) EM_total_DMmi≥(FE_Di·FC_Di·(dis_DMmi)·1.05)−M(1−x_DM_mir′)−M(x_DM_mit′),∀m∈M, ∀i∈I,

(95) e_u_DMmi≥((v·ρr·EM_total_DMmi)(LOmax⁡_Dir′·LF_Dir′))−M(1−x_DMmir′)−M(x_DMmir′), ∀m∈M,  ∀i∈I,

(96) EM_total_DMmi≥(10−3·(ξh·T·FER)106(W_gr·LF_Dit′))−M(1−x_DMmit′)−M(x_DMmir′), ∀m∈M,  ∀i∈I,

(97) e_u_DMmi≥(EM_total_DMmi·dis_DMmi·wp)−M(1−x_DMmit′)−M(x_DMmir′),∀m∈M, ∀i∈I.

Nonlinear equation (77) is the objective function which minimizes total cost of the carbon emissions allocated to whole units of the product for transportation from manufactory to DC, DC to customer, customer to customer, customer to DC, DC to dismantler, and dismantler to manufactory, respectively. Constraints (78)–(85) show the emissions allocated to one unit of the product for transportation from the *i*th manufactory to the *j*th DC, where *x*_MD_*ija*_ ⋯ *x*_MD_*ijw*_ are the binary variables to link carbon emissions constraints to the related types of transport. Constraints (78)-(79), (80)-(81), (82)-(83), and (84)-(85) measure carbon emissions of the aircraft, vehicle, diesel train, and vessel based on NTM method for air transport, road transport, rail transport, and water transport.

Constraints (86) and (87) show the emissions allocated to one unit of the product for transportation from the *j*th DC to the *k*th customer. Constraints (88) and (89) show the emissions allocated to one unit of the product for transportation from the *k*th customer to the *l*th customer. Constraints (90) and (91) show the emissions allocated to one unit of the product for transportation from the *k*th customer to the *j*th DC. Constraints (92) and (93) show the emissions allocated to one unit of the product for transportation from the *j*th DC to the *m*th dismantler. Constraints (86)–(93) measure carbon emissions of the vehicle based on NTM method for road transport. Constraints (94)–(97) show the emissions allocated to one unit of the product for transportation from the *m*th dismantler to the *i*th manufactory, where *x*_DM_*mir*′_ and *x*_DM_*mit*′_ are the binary variables to link carbon emissions constraints to the related types of transport. Constraints (94)-(95) and (96)-(97) measure carbon emissions of the vehicle and diesel train based on NTM method for road transport and rail transport.

(*1) Linearization.* To improve the performance of the proposed mathematical model we act out the following linearization for the nonlinear equations. As (77) is nonlinear, we turn it into the following equation and turn the constraints related to the definition of EM_total_MD_*ij*_,…, EM_total_DM_*mi*_ into the following equations:

(98) Equation  (77)⟶∑i∈I ∑j∈Je_u_MDij·CE+∑j∈J ∑k∈Ke_u_DCjk·CE+∑k∈K ∑l∈Ke_u_CCkl·CE+∑k∈K ∑j∈Je_u_CDkj·CE+∑j∈J ∑m∈Me_u_DDjm·CE+∑m∈M ∑i∈Ie_u_DMmi,

(99) Constraint(78)⟶EM_total_MDij≥y_MDija·(CEF+(VEF·0.801dis_MDij))−M(1−x_MDija)−M(x_MDijr+ x_MDijt+x_MDijw),∀i∈I, ∀j∈J,

(100) Constraint  (80)⟶EM_total_MDij≥y_MDijr·(FE_M·FC_M·(dis_MDij))−M(1−x_MDijr)−M(x_MDija+ x_MDijt+x_MDijw),∀i∈I, ∀j∈J,

(101) Constraint(82)⟶EM_total_MDij≥y_MDijt·(10−3·(ξf·T·FER)106(W_gr·LF_Mt))−M(1−x_MDijt)−M(x_MDija+ x_MDijr+x_MDijw),∀i∈I, ∀j∈J,

(102) Constraint(84)⟶EM_total_MDij≥y_MDijw·(FCW·FEW·1.2dis_MDij)−M(1−x_MDijw)−M(x_MDija+ x_MDijr+x_MDijt),∀i∈I, ∀j∈J,

(103) Constraint(86)⟶EM_total_DCjK≥y_DCjkvj·(FE_Dvj·FC_Dvj·(dis_DCjk))−M(1−x_DCjkvj),∀k∈K,  ∀j∈J, ∀vj∈VJ,

(104) Constraint  (88)⟶EM_total_CCKl≥y_CCklvj·(FE_Dvj·FC_Dvj·(dis_CCkl))−M(1−z_CCklvj),∀k,l∈K, ∀vj∈VJ,

(105) Constraint  (90)⟶EM_total_CDKj≥y_CDkjvj·(FE_Dvj·FC_Dvj·(dis_DCjk))−M(1−x_CDkjvj),∀k∈K,  ∀j∈J, ∀vj∈VJ,

(106) Constraint  (92)⟶EM_total_DDjm≥y_DDjmvj·(FE_Dvj·FC_Dvj·(dis_DDjm))−M(1−x_DDjmvj),∀m∈M,  ∀j∈J, ∀vj∈VJ,

(107) Constraint(94)⟶EM_total_DMmi≥y_DMmir′·(FE_Di·FC_Di·(dis_DMmi)· 1.05)−M(1−x_DMmir′)−M(x_DMmit′),∀m∈M, ∀i∈I,

(108) Constraint(96)⟶EM_total_DMmi≥y_DMmit′·(10−3·(ξh·T·FER)106(W_gr·LF_Dit′))−M(1−x_DMmit′)−M(x_DMmir′),∀m∈M, ∀i∈I,

#### 3.2.2. Emission-Constraint Model

This problem extends the basic model by constraining the carbon emissions, which is denoted by EM_Average (in Kg). We note that the constraint for carbon emissions is equal to the average of total carbon emissions for the basic model and emission cost-minimization problem. In this model, the objectives are the same as the basic model ones. Constraints (79), (81), (83), (85), (87), (89), (91), (93), (95), (97), (99)–(108) and the new one are added to the proposed basic closed-loop model to define and limit the carbon emissions issue. In order to formulate this emission-constraint model mathematically, the following notation is necessary.

*Parameters*

EM_Average: The average carbon emissions of the entire system.

The new constraint added to the emission cost-minimization model is as follows.

*The New Constraint*

Consider the following:

(109) ∑i∈I∑j∈Je_u_MDij+∑j∈J∑k∈Ke_u_DCjk  +∑k∈K∑l∈Ke_u_CCkl+∑k∈K∑j∈Je_u_CDkj  +∑j∈J∑m∈Me_u_DDjm  +∑m∈M∑i∈Ie_u_DMmi≤EM_Average.

The carbon emissions constraint is shown by Constraint (109).

## 4. Numerical Experiments

Here, we propose a numerical example to indicate the effectiveness of the proposed mathematical models. Our models are tested in small scale of data. Tables 1, 2, 3, 4, 5, 6, 7, 8, 9, and 10 are the given data. The numbers of potential locations for the manufactory, DC, and dismantler are three, four, and two, respectively. Manufactories, DCs, and dismantlers are selected to secure 57 customers having definite demands. While the applied optimization software is not able to provide solutions for 57 customers in a reasonable time, we categorized the customers into 7 more comprehensive zones with aggregated demands. There are four types of transportation mode (air, rail, road, and water) used to transfer product from manufactories to DCs; one type of transportation modes (road) is used to transfer product from DCs to customers and dismantlers, and two types of transportation modes (rail and road) are used to transfer product from dismantlers to manufactories.

For each of the four transport classes used to transfer product from manufactories to DCs, we select a representative vehicle to which we apply the NTM method. 

*Air Transport.* We select a cargo aircraft whose emission factors are most similar to the average values [28]. For the cargo aircraft we select the maximum load (LO_max⁡__*M*_*a*_) to be 29029 kg. We note that the distance over road (*D*_*r*_) between two locations is always more than the air distance (*D*_*a*_) and we find the following value *D*_*a*_ = 0.801 *D*_*r*_ on average in Google Maps [32].

*Road Transport.* We assumed that a semitrailer is used, because it is a common type to use for longer distance. The road type is supposed to be a motorway. We assume a load factor of 70%, which is typical for transport via integrating terminals [30]. The maximum load (LO_max⁡__*M*_*r*_) is 40 tonne.

*Rail Transport.* It is supposed that the rail network is designed for only diesel trains. All constants below are taken from NTM Rail [29]. We assume that the gross weight (*W*_gr) of the train is 1000 tonne, which is the average value specified by NTM Rail [29]. The entire track from manufactories to DCs is flat and we find the following value *ξ*_*f*_ = 1 in NTM Rail [29]. We assume that the rail distance between two locations is equal to the road distance. For a diesel train we take the following parameter values.

*Water Transport.* We assume that inland waterways are used for transport and that a general cargo vessel is used. For inland waterways NTM assumes a load factor of 50% [31]. The cargo capacity (maximum load) of a general cargo vessel for inland waterways is 1920 tonne. We assume that the distance between two locations over inland waterways is larger than the distance over road. The distance (*D*_*w*_) is therefore 1.2 times the road distance (*D*_*r*_). For a general cargo vessel we take the following parameter values.

For one type of transport modes used to transfer product from DCs to customers and dismantlers, we select two representative vehicles to which we apply the NTM method.

*Road Transport*. We assumed that two lorries are used: 5-tonne lorry and 40-tonne lorry. The road type is supposed to be a motorway. For two lorries we take the following parameter values.

For each of the two transport classes used to transfer product from dismantlers to manufactories, we select a representative vehicle to which we apply the NTM method.

*Road Transport.* We assumed that a semitrailer is used, because it is a common type to use for longer distance. The road type is supposed to be a hilly terrain. We assume a load factor of 50%. The maximum load (LO_max⁡__Di_*r*′_) is 40 tonne. To account for hilly terrain we add 5% [30] to the total emissions.

*Rail Transport.* It is supposed that the rail network is designed for only diesel trains. All constants below are taken from NTM Rail [29]. We assume that the gross weight (*W*_gr) of the train is 1000 tonne, which is the average value specified by NTM Rail [29]. The entire track from dismantlers to manufactories is hilly and we find the following value *ξ*_*h*_ = 1.25 in NTM Rail [29]. We assume that the rail distance between two locations is equal to the road distance. For a diesel train we take the following parameter values in Table 3.

There are five types of connection links in the proposed closed-loop chain. Possible connection links are as follows:

- connection link between the manufactory and DC,
- connection link between the DC and customer,
- connection link among customers,
- connection link between the DC and dismantler,
- connection link between the dismantler and manufactory.

Table 4 shows distances related to the defined connection links and transfer times between DCs and customers and among customers using a variety of vehicles. Maximum and minimum waiting time for customers are set to be 500 and 2500 units of time, respectively. Manufactory, distribution center, customer, and dismantler are involved with the respective numbers (capacity, demand, fixed cost, production cost, and rate) as shown in Table 5 and three rates are assumed to be different with respect to each DC, customer, and dismantler, respectively.  Table 6 lists the unit cost of transportation. The recovery costs in DC are given in Table 7 and are assumed to be equal for each DC with respect to each customer.  Table 8 shows the vehicle properties. The weight and volume of the product are assumed to be 40 (kg) and 0.5 (m^3^), respectively. The maximum (*Q*) and minimum (*L*) numbers of customers a salesman must visit are supposed to be 4 and 1, respectively. The fixed cost for landfilling is set to be €2 per unit. Since the establishment of the carbon market price has varied between €1 and €30/tonne, we consider the average cost of carbon emission (i.e., €15/tonne).

**Figure 2:**
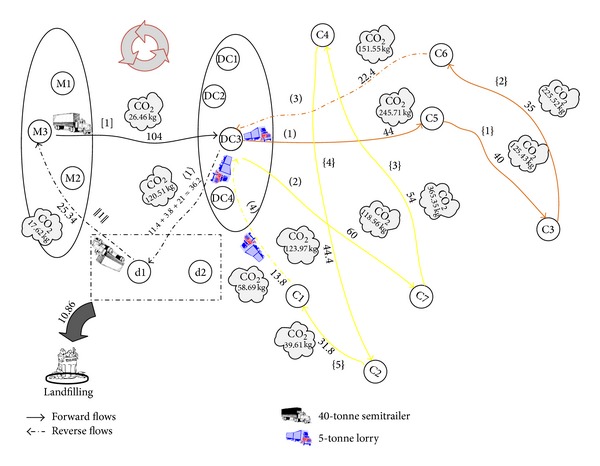
Optimal closed-loop chain of the numerical experiment without considering the carbon emission issue (basic model).

**Figure 3:**
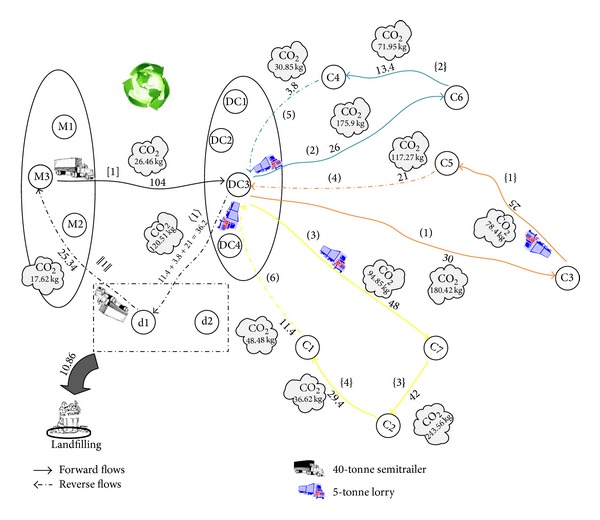
Optimal closed-loop chain of the numerical experiment with considering the carbon emission issue (emission cost-minimization model).

**Figure 4:**
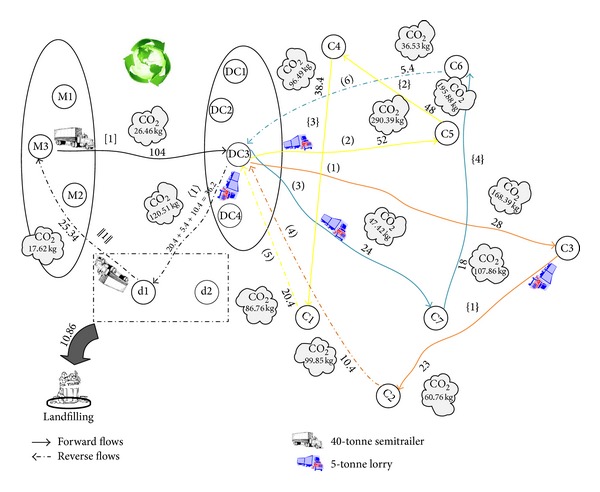
Optimal closed-loop chain of the numerical experiment with considering the carbon emission issue (emission-constraint model).

So far, we present the required data for processing the results. To facilitate the computations in our mixed integer programming (MIP) models, GAMS 22.9 software package is applied. The General Algebraic Modeling System (GAMS) is a high level modeling system for mathematical programming and optimization and is specifically designed for modeling linear, nonlinear, and mixed integer optimization problems. The system is especially useful with large and complex problems [33]. After solving the first two models (basic and emission cost-minimization models) using this software, we have found that the total carbon emissions for the basic and emission cost-minimization problems are 1618.98 (kg) and 1242.89 (kg), respectively. So, the average of these values as a constraint of carbon emissions (EM_Average) for the third model (emission-constraint model) is calculated to be (1618.98 + 1242.89)/2 = 1430.93≅1431 (kg). We have reported the results in Table 9 along with the optimal solution obtained for three cases, for comparison purposes. The validity of model is measured for numerical experiment as seen in Figures 2, 3, and 4, schematically. The results are summarized in Table 9. Table 9 presents objective function of three cases. As expected, the basic case has the best objective function value, that is, the lowest cost. The objective function values in the cost-based case and constraint-based case are little higher than the basic case. Product flow rates and amount of CO_2_ (kg) emitted from journeys of selective paths are shown in Table 9. There are five types of connection links in the selective path column.

- Links connecting between the manufactory and DC are indicated by *a*-*b*: [*n*] format, where *a* and *b* are numbers which indicate selective manufactory and DC, respectively. *n* is a number which indicates a selective path on the figure. [·] is a symbol related to this kind of connection links.
- Links connecting between the DC and customer are indicated by *c*-*d*: (*n*) format, where *c* and *d* are numbers which indicate selective DC and customer, and vice versa. (·) is a symbol related to this kind of connection links.
- Links connecting among the customers are indicated by *e*-*f*: {*n*} format, where *e* and *f* are numbers which indicate selective customers, respectively. {·} is a symbol related to this kind of connection links.
- Links connecting between the DC and dismantler are indicated by *g*-*h*: 〈*n*〉 format, where *g* and *h* are numbers which indicate selective DC and dismantlers, respectively. 〈·〉 is a symbol related to this kind of connection links.
- Links connecting between the dismantler and manufactory are indicated by *i*-*j*: ||*n*|| format, where *i* and *j* are numbers which indicate selective dismantler and manufactory, respectively. ||·|| is a symbol related to this kind of connection links.

From Table 9, the following is concluded.

1. For three cases, only one manufactory (number 3) and one DC (number 3) are selected to secure and transport the total sum of customers' demands. Besides, only one dismantler (number 1) is selected to transport the recovered product to the manufactory.
2. For three cases, one route exits from the manufactory (number 3).
3. The aggregate value of product flow in exiting paths from a manufactory is equivalent to the total sum of customers' demands. That means that all of customers' demands in the network are met.
4. The value of product flow in exiting path from a DC is equivalent to the total sum of demands of customers which belongs to the same tour. That means that all of customers' demands in each tour are met.
5. The value of product flow in exiting path from a customer is equivalent to the total sum of demands of remaining customers which belongs to the same tour plus the amount of recovered product obtained from customer. For example, corresponding to Figure 2, the value of product flow in exiting path from a customer (number 5) is calculated to be 10 + 14 + (0.8 × 20) = 40 units where customer's (numbers 3, 6, and 5) demands and the percentage of recovery of customer (number 5) are 10, 14, 20, and 80%, respectively.
6. In the reverse flow, the aggregate value of returned product flow in exiting paths from a DC is equivalent to the total sum of customers' demands of recovered product. For example, corresponding to Figure 2, this value is calculated to be 11.4 + 3.8 + 21 = 36.2.

For basic, cost-based, and constraint-based cases, an optimal closed-loop chain is shown in Figures 2, 3, and 4, respectively. In these figures, we consider a particular color for each tour in which a salesman departs from selective DCs and arrives to the customers. So, the selective path given in Table 9 is indicated by different colors. The objective functions for basic, cost-based, and constraint-based cases are 230023.6, 233765.9, and 231159 units in 1886, 1380, and 4610 seconds, respectively. Note that this computation time is needed to be spent for solving a problem with three, four, and two potential locations for the manufactory, DC, and dismantler and seven customers. The suitable paths to deliver product to customers from manufactories and DCs in the forward flows, to deliver recovered product to dismantlers from DCs and customers, and to deliver reused product to manufactories from dismantlers in the reverse flows for basic, emission cost-minimization, and emission-constraint models are shown in Figures 2, 3, and 4. In addition, the selected vehicles for carrying product and the corresponding amount of product are illustrated in them which also include the amount of CO_2_ (kg) emitted from journeys and the amount of landfills. The arrival time of product at each customer for three models is reported in Table 10. The traffic light that turned green in all shows that the time window of each customer is satisfied and the arrival time of product is within the allowed range ([500–2500]). The objective values of the three cases (i.e., basic, cost-based, and constraint-based cases) are very close to each other. Therefore to verify the differences, we work out a sensitivity analysis given in Section 5.

## 5. Comparison of the Three Closed-Loop Supply Chain Models

**Figure 5:**
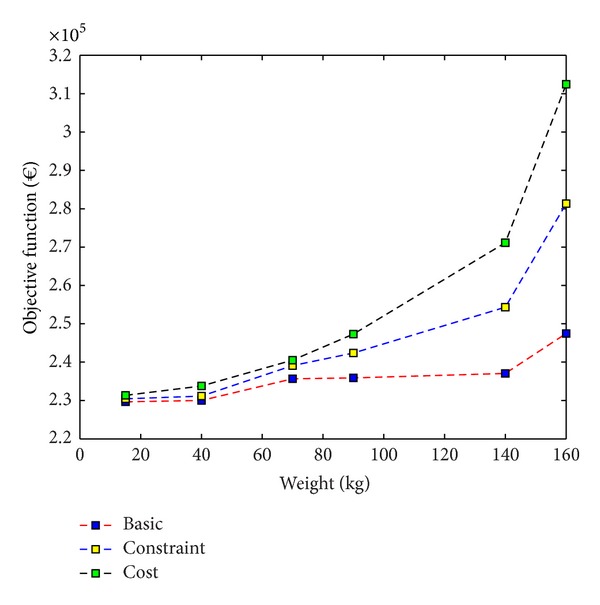
The objective value for the basic, cost-based, and constraint-based models.

Here, we investigate the variation of the total costs and CO_2_ emissions in different product's weights obtained from all three cases. First, we consider the total cost obtained from basic, cost-based, and constraint-based approaches. We depict the total costs for different product's weights in Figure 5. Since the feasible region of the basic model is larger than or equal to the feasible regions of the cost-based and constraint-based models, the optimal value of the former is no worse than the optimal value of the latter. This implies that the optimal value to the basic model is a lower bound on the optimal value for the problems where we integrated the environmental issues into a traditional logistic system. Thus, the minimum objective value of the solution in the feasible region of the basic model will be less than or equal to the two others. Moreover, increasing the weight of the product leads to high total costs due to the vehicle capacity constraints. Here, we survey the CO_2_ emissions obtained as output of the proposed mathematical model.

**Figure 6:**
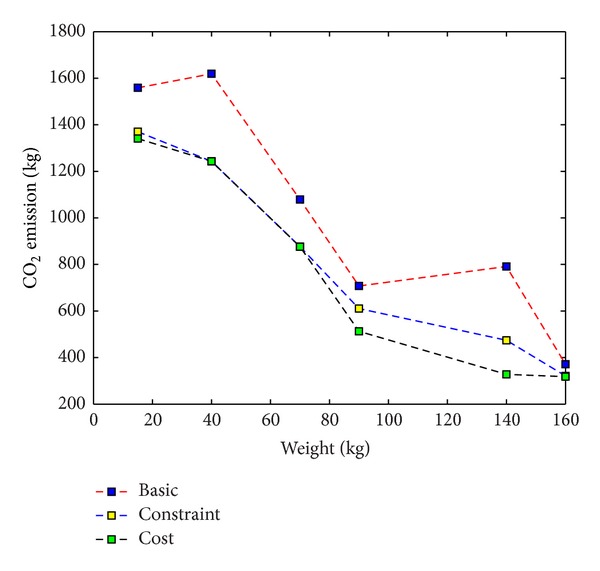
The CO_2_ emissions for the basic, cost-based, and constraint-based models.

We see in Figure 6 that the basic model has the highest carbon footprint among the three scenarios due to ignorance of this issue in its assumptions. Thus, the optimal values for emissions of the cost-based and constraint-based models which both consider the carbon footprint are no worse than the value of the basic model. This implies that the emission value to the basic model is an upper bound on the optimal value for the two others. So, the emissions value of the basic model solution will be more than the two others. With the increase of product's weight, the carbon emission is decreasing. This result confirms the finding in [8] that the following ordering for unit emissions is

(110) $$\begin{array}{c} e\_u_{\text{air}}>e\_u_{\text{road}}>e\_u_{\text{rail}}>e\_u_{\text{water}}. \end{array}$$

Due to the vehicle capacity constraints, the larger vehicles are responsible to carry one commodity from a location to another. For the two cases which consider carbon footprint value in their calculations, the models are more likely to choose larger vehicles such as train and cargo vessel. This is not necessarily because of their capacity to carry heavy loads or time element, since the air transport excels at doing so, but because of the emission element. So, as we can see in Figures 5 and 6, with the increase of product's weight, the use of these vehicles is likely and leads to the increase in costs and the decrease in emission intensity.

**Figure 7:**
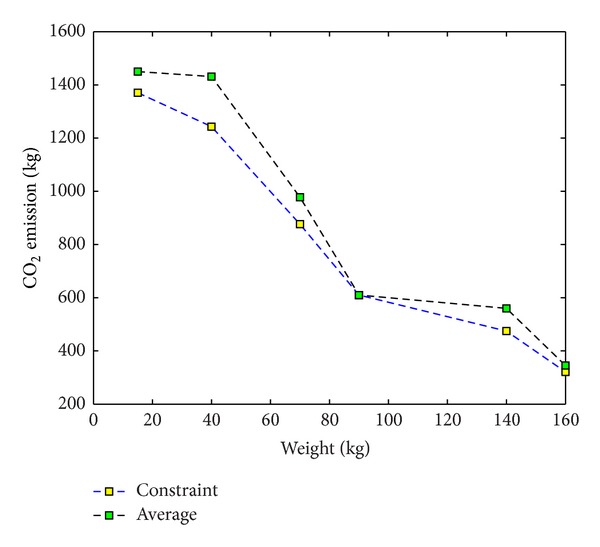
The comparison between the emission decision variable and EM_Average for the third problem.

Although the basic model is less expensive than other models, it is not a complete model because it does not attempt to consider the carbon emission issue. The upper bound of emission intensity to calculate an emission restriction for constraint-based model is just as it used to be. Then, we will just focus on the other two models. Clearly, with respect to the definition of EM_Average for the third problem, this method is between the other two methods in carbon emissions. These quantities in constraint-based and cost-based methods are closer than basic method. Because the cost-based approach necessitates a model to adopt environmentally friendly vehicles such as train and cargo vessel, you can expect the total cost estimate to be the highest value. In contrast, the constraint-based approach has more freedom to choose the vehicles that are likely to be cheaper and not greener and safer for the environment. Consequently, the total cost is reduced by 4.6% and the CO_2_ emission is increased by 5.9%. Changes in emission intensities obtained from solving the third model over product's weight are compared with the certain amount of EM_Average and shown in Figure 7.

Thus, we find that the performance of constraint-based model is better than cost-based approach considering the total costs and CO_2_ emission decision variables.

## 6. Conclusions

In this work, we presented an extended closed-loop supply chain network to integrate the environmental issues into a traditional logistic system. Our proposed chain contained four layers (manufacturers, DCs, customers, and dismantlers). Finding optimal locations of manufacturers, DCs, and dismantlers and distribution of product satisfying time windows were our purposes that are attained in a mixed integer linear programming approach. In this way, we proposed an approach as multiple DCs multiple traveling salesman problem (MDMTSP) between DCs and customers. In addition to managing properly reverse logistics to reduce negative impact of greenhouse gases emissions, we focused on transport mode selection as a way to reduce emissions. For this, two types of regulations to reduce carbon emissions coming from freight transport were considered. The first mechanism specified a cost for carbon emissions and the second one was a constraint on emissions. Consequently, three models were formulated corresponding to these regulations and the effects of the regulations on the preferred transport mode and the emissions were investigated.

The applicability and effectiveness of our proposed model were tested through numerical example. Also, comparative analysis was investigated on decision variables.

## Figures and Tables

**Table 1:** Emission factors for representative vehicle from manufactories to DCs.

Cargo aircraft		Semitrailer (40 tonne)		Diesel train		Cargo vessel	
Load factor (%) (LF_*M*_*a*_)	80	Load factor (%) (LF_*M*_*r*_)	70	Load factor (%) (LF_*M*_*t*_)	50	Load factor (%) (LF_*M*_*w*_)	50
CEF (kg)	4139.6	FC (L/km) (FC_*M*)	0.3198	*T*	122.46	FEW (kg/tonne)	3178
VEF (kg)	15.353	FE (kg/L) (FE_*M*)	2.621	FER (g/L)	3175	FCW (tonne/km)	0.007

**Table 2:** Emission factors for representative vehicle from DCs to customers and dismantlers.

	Five-tonne lorry	40-tonne lorry
Load factor (%) (LF_*D*_*v*_*j*__)	75	90
FC_*D*_*v*_*j*__ (L/km)	0.245	0.369
FE_*D*_*v*_*j*__ (kg/L)	2.63	2.63

**Table 3:** Emission factors for representative vehicle from dismantlers to manufactories.

Semitrailer (40 tonne)		Diesel train	
Load factor (%) (LF_Di_*r*′_)	50	Load factor (%) (LF_Di_*t*′_)	50
FC (L/km) (FC_Di)	0.293	*T*	122.46
FE (kg/L) (FE_Di)	2.621	FER (g/L)	3175

**Table 4:** Distance and transfer times.

DC	Distance	Transfer times using 5-tonne lorry	Transfer times using 40-tonne lorry	Distance	Transfer times using 5-tonne lorry	Transfer times using 40-tonne lorry	Distance	Transfer times using 5-tonne lorry	Transfer times using 40-tonne lorry	Distance	Transfer times using 5-tonne lorry	Transfer times using 40-tonne lorry	Distance	Transfer times using 5-tonne lorry	Transfer times using 40-tonne lorry	Distance	Transfer times using 5-tonne lorry	Transfer times using 40-tonne lorry	Distance	Transfer times using 5-tonne lorry	Transfer times using 40-tonne lorry
	Customers																				
	1			2			3			4			5			6			7		
1	85	500	800	149	380	430	75	780	1020	123	270	560	174	660	900	100	984	1329	250	654	780
2	328	300	450	187	280	403	98	857	1220	257	370	460	400	720	950	278	284	629	189	354	870
3	198	1500	1850	447	850	1430	280	780	1470	378	670	1060	260	760	987	315	1084	1629	92	947	1280
4	270	200	705	360	480	830	200	1180	1780	420	770	960	338	1050	1800	173	884	1330	290	554	856

	Customer-customer																				
1	0	0	0	58	180	203	128	65	120	117	37	60	68	72	95	150	28	75	249	35	87
2	58	180	203	0	0	0	123	78	147	130	67	106	88	76	98	125	108	162	270	94	128
3	128	65	120	123	78	147	0	0	0	317	77	96	146	105	180	300	88	133	210	55	85
4	117	37	60	130	67	106	317	77	96	0	0	0	190	66	90	250	98	132	315	65	78
5	68	72	95	88	76	98	146	105	180	190	66	90	0	0	0	190	67	97	320	57	110
6	150	28	75	125	108	162	300	88	133	250	98	132	190	67	97	0	0	0	279	85	111
7	249	35	87	270	94	128	210	55	85	315	65	78	320	57	110	279	85	111	0	0	0

DC	Manufactories									Dismantlers						Manufactories-dismantlers					
	1			2			3			1			2			1			2		

1	75	—	—	38	—	—	54	—	—	50	—	—	88	—	—	168	—	—	90	—	—
2	50	—	—	46	—	—	36	—	—	110	—	—	90	—	—	200	—	—	178	—	—
3	24	—	—	25	—	—	68	—	—	155	—	—	182	—	—	138	—	—	119	—	—
4	17	—	—	63	—	—	96	—	—	122	—	—	80	—	—						

**Table 5:** Capacity, demand, fixed cost, production cost, and rate.

Manufactory			DC			Customer		Dismantler		
Capacity (Cm)	Fixed cost (€) (FM)	Pro. cost (*€*) (P_cost)	Total capacity (Tc)	Fixed cost (*€*) (FDC)	Pc (%)	Demand (dc)	pr (%)	Capacity (Cd)	Fixed cost (*€*) (FD)	Pl (%)
1000000	200000	326	3000	80000	40	20	10	1600	20000	30
1000000	180000	400	5000	50000	20	18	30	2400	25000	38
1000000	150000	300	1500	23000	50	10	50			
			2000	30000	50	12	20			
						20	80			
						14	10			
						10	40			

**Table 6:** Unit cost (*€*) of transportation per km.

	DC			
	Cargo aircraft	Semitrailer	Diesel train	Cargo vessel
Manufactory	0.25	0.16	0.2	0.3

	Customer and dismantler			
	Five-tonne lorry		40-tonne lorry	

DC	0.13		0.18	

	Manufactory			
	Semitrailer		Diesel train	

Dismantler	0.15		0.25	

**Table 7:** The recovery cost (*€*) in DC from customer.

Rc	1	2	3	4
1	1.5	2.3	1.8	0.8
2	1.5	2.3	1.8	0.8
3	1.5	2.3	1.8	0.8
4	1.5	2.3	1.8	0.8
5	1.5	2.3	1.8	0.8
6	1.5	2.3	1.8	0.8
7	1.5	2.3	1.8	0.8

**Table 8:** The vehicles' properties.

Vehicle	Number of vehicles (unit) Manufactory (NVM) 1 2 3	Maximum load (kg) (LO_max⁡__*M*)	Capacity (kg) (CVM)	Density of product (*ρ*)
Cargo aircraft	2 1 2	29029	29029	167
Semitrailer	5 3 6	40000	40000	250
Diesel train	1 0 1	1000000	1000000	—
Cargo vessel	3 2 2	1920000	1920000	—

	DC (NVD)	(LO_max⁡__*D*)	(CVD)	
	1 2 3 4			

Five-tonne lorry	5 3 10 4	5000	5000	250
40-tonne lorry	3 6 6 1	40000	40000	250

	Dismantler (NVDi)	(LO_max⁡__Di)	(CVDi)	
	1 2			

Semitrailer	4 3	40000	40000	250
Cargo vessel	1 2	1000000	1000000	—

**Table 9:** Results for three cases.

Basic model			Emission cost minimization			Emission-constraint model		
Selective path	Amount of product flow	Amount of CO_2_ (kg)	Selective path	Amount of product flow	Amount of CO_2_ (kg)	Selective path	Amount of product flow	Amount of CO_2_ (kg)
3-3: [1]	104	26.46	3-3: [1]	104	26.46	3-3: [1]	104	26.46
3-5: (1)	44	245.71	3-3: (1)	30	180.42	3-3: (1)	28	168.39
3-7: (2)	60	118.56	3-6: (2)	26	175.9	3-5: (2)	52	290.39
5-3: {1}	40	125.43	3-7: (3)	48	94.85	3-7: (3)	24	47.42
3-6: {2}	35	225.52	3-5: {1}	25	78.4	3-2: {1}	23	60.76
7-4: {3}	54	365.35	6-4: {2}	13.4	71.95	5-4: {2}	48	195.88
4-2: {4}	44.4	123.97	7-2: {3}	42	243.56	4-1: {3}	38.4	96.49
2-1: {5}	31.8	39.61	2-1: {4}	29.4	36.62	7-6: {4}	18	107.86
6-3: (3)	22.4	151.55	5-3: (4)	21	117.27	2-3: (4)	10.4	99.85
1-3: (4)	13.8	58.69	4-3: (5)	3.8	30.85	1-3: (5)	20.4	86.76
3-1: 〈1〉	36.2	120.51	1-3: (6)	11.4	48.48	6-3: (6)	5.4	36.53
1-3: ||1||	25.34	17.62	3-1: 〈1〉	36.2	120.51	3-1: 〈1〉	36.2	120.51
			1-3: ||1||	25.34	17.62	1-3: ||1||	25.34	17.62

Landfill	10.86	—	Landfill	10.86	—	Landfill	10.86	—

Objective			Objective			Objective		

230023.6			233765.9			231159		

**Table 10:** The time windows.

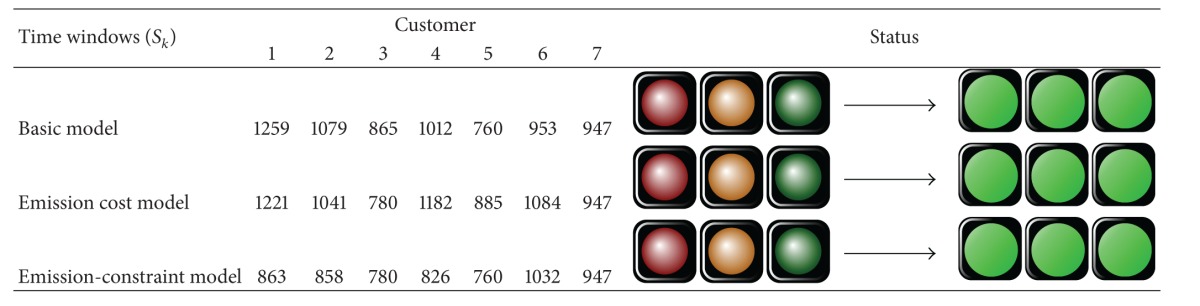
